# Risk assessment of antibiotic residues and resistance profile of *E*. *coli* in typical rivers of Sichuan, China

**DOI:** 10.1371/journal.pone.0306161

**Published:** 2025-02-11

**Authors:** Jingzhou Sha, Minghao Wu, Yaliang Zhou, Tao Cheng, Haisha Liu, Jingjing Zhang, Wan Luo, Yi Huang, Yinshan Liu, Baoming Wang, Tao Song, Jiafu Lin

**Affiliations:** 1 Sichuan Solid Waste and Chemical Management Center, Department of Ecology and Environment of Sichuan Province, Chengdu, Sichuan, China; 2 Department of Pharmacy, The First Hospital of Liangshan, Liangshan, Sichuan, China; 3 Sichuan University West China Hospital, Chengdu, Sichuan, China; 4 Antibiotics Research and Re-evaluation Key Laboratory of Sichuan Province, Sichuan Industrial Institute of Antibiotics, School of pharmacy, Chengdu University, Chengdu, Sichuan, China; Kampala International University - Western Campus, UGANDA

## Abstract

The presence and distribution of antibiotics and antibiotic resistance genes (ARGs) in rivers have attracted significant global concern. However, research on the contamination of typical rivers in Sichuan Province, China, remains limited. This study aimed to assess the residual levels of antibiotics across 42 national and provincial monitoring sites in nine rivers within Sichuan using UPLC-MS/MS. Ecological risk levels were evaluated through established risk assessment methods, and antibiotic resistance in *Escherichia coli*(*E*.*coli*) isolated from these waters was determined using the Kirby-Bauer disk diffusion method. Additionally, redundancy analysis (RDA) was conducted to explore the impact of residual antibiotics on the microbial community structure in the Minjiang River basin. Antibiotics were detected in all nine rivers studied, with the Minjiang, Tuojiang, and Jialingjiang rivers exhibiting particularly severe contamination, with concentrations ranging from 0.29 to 2233.71 ng/L. The level of antibiotic pollution in the Sichuan Basin was significantly higher than in other regions of Sichuan, likely due to the area’s high population density. Furthermore, 9.77% of *E*. *coli* isolates from the nine rivers exhibited antibiotic resistance, with over 5.8% demonstrating multidrugs resistance. Norfloxacin, amoxicillin, ampicillin, and tetracycline were identified as the primary contributors to the high ecological risk at 26 of the 42 monitoring sites. A strong correlation was observed between residual antibiotics and changes in microbial community structure. These findings provide critical insights into the distribution of antibiotics and ARGs in the rivers of Sichuan Province and highlight the urgent need for targeted strategies to mitigate antibiotic pollution. Addressing this issue is essential to protect both ecological integrity and public health.

## Introduction

Antibiotics have been extensively used in livestock and aquaculture for treating infections, preventing disease, and promoting growth [1, 2]. In 2010, global antibiotic consumption in these sectors was approximately 63,151 tons, with projections indicating a 67% increase by 2030 [3]. In the United States, 80% of the annual antimicrobial consumption is attributed to livestock feed [4]. Additionally, a substantial quantity of antibiotics is employed to sustain animal productivity in low- and middle-income countries, particularly in Brazil [5], Russia [6], India [7] and China [8], driven by the increasing demand for animal protein. As the consequence, the continued misuse of antibiotics has resulted in 50–80% of these substances entering the environment due to their persistence and low degradability [9, 10].

The presence of multiple antibiotics, such as oxytetracycline, chlortetracycline, tetracycline, and amoxicillin, has been documented in environments worldwide [11]. The gradual accumulation of these antibiotics in the environment fosters the binding, transmission, and gene transfer of ARGs, contributing to the emergence of drug-resistant pathogens and the subsequent failure of antibiotic treatments [12, 13]. Alarmingly, the European Centre for Disease Prevention and Control estimates that approximately 25,000 Europeans die annually from drug-resistant bacterial infections [14]. Similarly, the British government reports that over half a million deaths are attributable to such infections globally [15].

Water systems serve as a critical reservoir for antibiotics and ARGs [16, 17]. The accumulation of these substances in aquatic environments can exert long-term selective pressure, leading to reduced microbial diversity, alterations in microbial community structure, and further contamination of water resources [18, 19]. Consequently, the bioaccumulation of antibiotics in poultry, aquatic products, and vegetables poses a direct or indirect threat to human health through the food chain. For instance, a study detected eleven antibiotics, with a total concentration of 229 ng/L, in the drinking water source of the Yangtze River in China [20]. Additionally, fifteen types of antibiotics, including tetracyclines (TCs) and quinolones, were found in the surface water of the Yellow River [21].

Sichuan, China’s fifth most populous province, plays a key role in the country’s demographic landscape. In addition to its large population, past studies have shown that Sichuan ranks among the top provinces for livestock and swine populations, surpassing most other regions [22]. This high concentration of livestock highlights the need for focused research on antibiotic pollution in the province, particularly given the potential environmental impact of such a dense agricultural presence. Despite this, research on the concentration of antibiotic residues in river water, associated risk assessments, and the impact on aquatic microorganisms in Sichuan Province remains limited. Previous studies conducted in 2018 by Yang et al. and Tuo et al. identified ARGs in the Funan River in Sichuan, but these investigations were confined to a single river, limiting their ability to represent the overall extent of antibiotic pollution in Sichuan’s rivers [23, 24].

This study is the first to systematically investigate the concentration and ecological risk of antibiotic residues across typical water systems in Sichuan, encompassing nine rivers. Furthermore, it explores the level of *E*. *coli* resistance in these waters and examines the impact of antibiotic residues on the microbial community structure. The findings from this study will provide a foundational assessment of antibiotic pollution in Sichuan’s rivers and inform policymakers in developing effective strategies to reduce antibiotics pollution.

## Materials and methods

### Study sampling-sites and sample collection

In this study, a total of 1–13 sampling sites were established across nine major rivers in Sichuan Province: the Minjiang River, FuJiang River, TuoJiang River, JiaLingJiang River, QuJiang River, DaDuHe River, YaLongJiang River, JinShaJiang River, and HuangHe River. The selection of sampling sites was based on the official Chinese government document, the "13th Five-Year Plan (2016–2020) for Surface Water Environmental Quality Monitoring Site Distribution Map of Sichuan Province," which identifies provincial and national control assessment monitoring points. To ensure sample uniformity, water samples from the left bank, middle, and right bank of the same monitoring site were mixed (Table 1). These sites were distributed across 17 prefecture-level cities and 2 autonomous prefectures and the sampling was conducted from April to September 2021 to account for these seasonal differences. During the sample collection process, additional data on population distribution, industrial layout, and hydrological conditions around the sampling points were also gathered (Table 2).

**Table 1:** Main information of typical rivers in Sichuan.

River	Steam length(km)	Catchment (10^3^km^2^)
Minjiang River	1279	133.5
FuJiang River	700	36.4
TuoJiang River	712	27.9
JiaLingJiang River	1345	160
QuJiang River	720	40.5
DaDuHe River	1050	77.7
YaLongJiang River	1357	136
JinShaJiang River	3481	502
HuangHe River	5464	752.4

**Table 2:** Scale of poultry breeding and animal husbandry in Sichuan Province.

Year	Livestock and Husbandry industry			aquaculture industry
	Swine(number)	Cattle and sheep (number)	Poultry(number)	Aquatic products(t)
2021	6.31×10^7^	2.05×10^7^	7.74×10^8^	1.66×10^6^
2020	5.61×10^7^	2.08×10^7^	7.74×10^8^	1.60×10^6^
2019	4.85×10^7^	2.07×10^7^	7.87×10^8^	1.57×10^6^
2018	6.63×10^7^	2.01×10^7^	6.60×10^8^	1.53×10^6^
2017	6.57×10^7^	2.04×10^7^	6.52×10^8^	1.54×10^6^
2016	6.92×10^7^	2.06×10^7^	6.77×10^8^	1.45×10^6^

### Sample collection and processing

A total of 5 liters of water was collected for antibiotic residue analysis. Since the antibiotic determination in this study was conducted using the solid-phase extraction UPLC-MS/MS method, the required water volume typically ranges between 2–3 liters for extraction. For the analysis of environmental microbial diversity, 16S-DNA high-throughput sequencing was employed, which generally requires 500 mL of water for total environmental DNA extraction. Additionally, 50 mL of water samples were used for the isolation and screening of Enterobacteriaceae bacteria. Water samples were collected using sterile polyethylene containers, and a professional water sampler (Water sampler, BC-S-1000, Boruite technology Co., Ltd, FuShun, China) was employed for the collection process.

### Chemical reagents/solvents and target antibiotics

In this study, 15 types of antibiotics across 6 categories were analyzed which were those most commonly used in Sichuan Province’s livestock and poultry farming enterprises, as well as in the medical field [25]. These included beta-lactams (amoxicillin [AMP], ampicillin [AMX], cephalexin [CEX], cefotaxime [CTX]), quinolones (enrofloxacin [ENR], levofloxacin [LEV], norfloxacin [NOR], moxifloxacin [MOX]), sulfonamides (sulfadiazine [SD], sulfamethoxazole [SMZ]), tetracyclines (oxytetracycline [OTC], tetracycline [TE], chlortetracycline [CTC]), amide alcohols (chloramphenicol [CAP]), and lincosamides (clindamycin [CLI]). These antibiotics were procured from the China Institute for Food and Drug Control. All solvents and chemical reagents used were either analytically pure or chromatographically pure. Internal standards, including C13-caffeine, amoxicillin-D4, ciprofloxacin-D8, sulfamethoxazole-D4, thiabendazole-D4, and chloramphenicol-D5, were also purchased from the China Institute for Food and Drug Control.

### Antibiotics analysis

For the pre-treatment of samples, 500 mL of water was filtered using a fiberglass filter membrane (20191209001, NEWSTAR, China). To this, 0.5 g Na2EDTA and 100 ng of a mixed internal standard were added as a recovery indicator, and the pH was adjusted to 3.0 using 4M sulfuric acid. Solid-phase extraction (SPE) was employed to concentrate the antibiotics: the water sample was loaded onto an activated HLB column(WAT094226, Waters, USA) at an extraction flow rate of 6–8 mL/min. The container was then rinsed with 25 mL of 5% methanol water (Vmethanol: Vwater), and the washing solution was added to the HLB column (500 mg, 6 mL). Finally, the HLB column was washed with 10 mL of ultrapure water to remove Na2EDTA and other residual impurities. The HLB column was dried under vacuum for 120 minutes, and the target substances were eluted with 8 mL of methanol. The volume of the target substances was concentrated to 1 mL by adding 60% methanol water (Vmethanol: Vwater). The sample solution was filtered through a 0.22 μm filter membrane (20191209001, NEWSTAR, China), stored in a brown bottle at -20°C, and analyzed using UPLC-MS/MS (UPLC: A-30, Japan; MS/MS: ABSciex Qtrap3200, Agilent, USA).

The mass spectrometry conditions were optimized, including ion source mode, cone voltage, parent ion, collision energy, and product ions. The chromatographic column used was Athena C18-WP (2.1×150 mm, 3 μm); the injection volume was 5 μL, with a mobile phase flow rate of 0.5 mL/min, and the column temperature was maintained at 40 degrees. Mobile phase A was 0.1% formic acid, while mobile phase B was acetonitrile. Chloramphenicol was analyzed in multiple reaction monitoring [NRM] negative ion mode, while the other 14 antibiotics were analyzed in MRM positive ion mode. The UPLC-MS/MS operated in multiple reaction monitoring mode with an Electro-spray Ionization ESI ion source, with drying gas temperature and flow rate set at 325°C and 6 mL/min, respectively. The sheath gas flow rate was 11 L/min, the ionization voltage was 2.5 kV, and the atomizer pressure was 45 psi.

### Sequencing

After water samples from the Minjiang River Basin were collected, they were transported to the laboratory at 4°C. The water samples were filtered using a 0.22 μm microporous membrane (20191209001, NEWSTAR, China) to collect microorganisms from the water, after which the total environmental microbial DNA was extracted. The extracted DNA samples were then used for sequencing. The primers and type of sequencing had provided in the method. The sequencing method has been provided below: the universal primers S-D-Bact-0341-bS-17 (5’-CCT ACG GGN GGC WGC AG-3’) and S-D-Bact-0785- a-A-21 (5’-GAC TAC HVG GGT ATC TAA TCC-3’), targeting the hypervariable V3/V4 region of the 16S rRNA gene were used as described by Klindworth et al [26]. All PCR reactions were carried out with Phusion® High-Fidelity PCR Master Mix (New England Biolabs). Sequencing libraries were generated using TruSeq® DNA PCR-Free Sample Preparation Kit (Illumina, USA) following manufacturer’s recommendations, and index codes were added. The library quality was assessed on the Qubit@ 2.0 Fluorometer (Thermo Scientific) and Agilent Bioanalyzer 2100 system. Finally, the library was sequenced on an IlluminaHiSeq2500 platform and 250-bp paired-end reads were generated.

All data processing was performed using the Quantitative Insights into Microbial Ecology 2 (QIIME 2) and R Statistical Software Version 3.44. The R package phyloseq was used to generate bar charts depicting relative and absolute abundance of taxa [27].

Sequence analysis were performed by Uparse software (Uparse v7.0.1001, http://drive5.com/uparse/) [28]. Sequences with ≥97% similarity were assigned to the same OTUs. Representative sequence for each OTU was screened for further annotation.

OTUs abundance information were normalized using a standard of sequence number corresponding to the sample with the least sequences. Subsequent analysis of alpha diversity and beta diversity were all performed basing on this output normalized data. In order to evaluate the shared microbial population and relative microbial abundance in each study site, venn diagram and bar graph were performed in R software.

Redundancy analysis (RDA) was utilized to analyze the relationship between antibiotics residual and bacterial communities. RDA was performed in R software. Alpha diversity is applied in analyzing complexity of species diversity for a sample through 4 indices, including species value, Chao1value, Shannon value, Simpson value. All these indices in our samples were calculated with QIIME2 and displayed with R software.

### Environmental risk assessment

The ecological risk quotient (RQE_cotox_) of antibiotic residues in the environment was evaluated using the risk quotient method, based on EU (European Union) risk assessment technical guidance. The formula used was RQE_cotox_ = MEC/PNECE_cotox_, where MEC is the measured environmental concentration of pollutants, and PNECE_cotox_ is the predicted no-effect concentration, representing the toxicological data (Table 3) of the most sensitive species to the target antibiotics. An RQE_cotox_ < 0.1 indicates a low-risk grade, RQE_cotox_ < 1 indicates a medium-risk grade, and RQE_cotox_ ≥ 1 indicates a high-risk grade.

**Table 3:** The PENC_Ecotox_ for risk assessment.

Antibiotic	Species	Type of Toxicity	Critical effects	Toxic Date (μg/L)	Evaluation factors(AF)	PENC (μg/L)
OTC	Algae	acute	-	-	-	0.23
TE	Algae	acute	EC20	-	-	0.0605
CTC	Algae	acute	EC50	50	1000	0.05
LCEX	Algae	acute	EC50	1000	1000	1
AMP	Algae	acute	NOEC	0.031	100	0.00031
AMX	Algae	acute	EC50	3.7	1000	0.0037
CTX	Algae	acute	LOEC	210	1000	0.21
SMZ	Algae	acute	NOEC	1	100	0.01
SD	Algae	acute	EC50	135	1000	0.135
LEV	water flea	acute	-	340	1000	0.34
ENR	Algae	acute	EC50	49	1000	0.049
NOR	Algae	acute	NOEC	20	1000	0.02
MOX	Algae	acute	-	340	1000	0.34
CLI	Algae	acute	EC50	14	1000	0.014
CAP	Bacteria	acute	EC50	64.3	1000	0.0643

Note: TCs, Tetracycline; OTC, oxytetracycline; CTC, chlortetracycline; TE, tetracycline; AMP, amoxicillin; AMX, ampicillin; CEX, cephalexin; CTX, cefotaxime; SAs, Sulfonamides; SD, sulfadiazine; SMZ, Sulfamethoxazole; FQs, Quinolones; ENR, enrofloxacin; LEV, levofloxacin; NOR, norfloxacin; MOX, moxifloxacin; LINs, Lincomide; CLI, clindamycin; CRPs, Amide alcohols; CAP, chloramphenicol.

### Screening/identification of Enterobacter and analysis of bacterial drug resistance

*E*.*coli* was isolated and screened based on the method reported by Ibrahim et al [29]. A 0.1 mL sample of river water was evenly spread on MacConkey agar medium for culture, and typical *E*. *coli* colonies (brick red) were selected for purification. The purified bacteria were stored and subjected to subsequent biochemical tests, including fermentation, oxidase, and indole tests. Strains that tested positive for fermentation, negative for oxidase, and positive for indole were selected out. Next, the 16S rDNA gene fragment was amplified, and the sequences were analyzed using NCBI BLAST for confirmation. E. coli strains matching the identification results were preserved for further analysis.

The sensitivity of *E*. *coli* to four antibiotics—gentamicin (10 μg), tetracycline (30 μg), levofloxacin (5 μg), and ofloxacin (5 μg)—was analyzed using the agar diffusion method (K-B method). The susceptibility of Enterobacteriaceae to antibiotics was classified as susceptible (S), intermediate (I), or resistant (R), according to the Clinical and Laboratory Institute implementation standards (2019 version).

### Statistics

All statistical analyses were performed using SPSS 19.0 software. Differences between groups were analyzed using one-way ANOVA followed by Tukey’s post hoc test for multiple comparisons. Results are expressed as mean ± standard deviation (SD), unless otherwise stated.

### Ethics statement

All field surveys were conducted in public rivers of the People’s Republic of China in accordance with the Order of the President of the People’s Republic of China (No. 74, 2002) and the Sichuan Water Resources Regulations (Announcement No. 110 by the Standing Committee of the 13th People’s Congress of Sichuan Province, 2021). No protected species were involved in this study. The field research protocol was approved by the Sichuan Provincial Department of Ecology and Environment. As this study focused on antibiotics and their resistance genes, it did not involve human or animal subjects and was therefore not subject to review by an institutional review board.

## Results

### Analysis of residual characteristics of antibiotics in typical rivers of Sichuan

**Fig 1:**
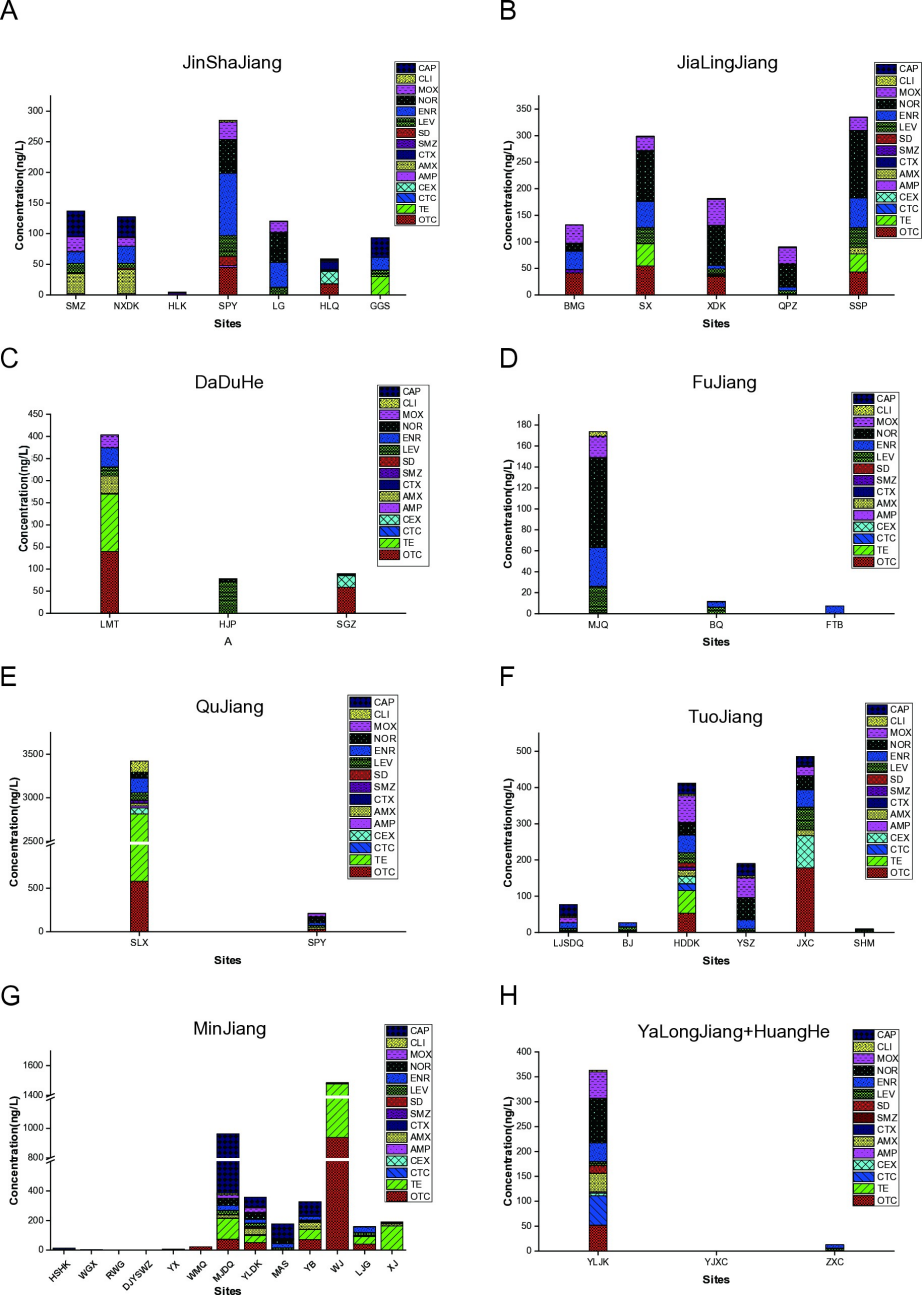
Antibiotics concentration at monitoring sites in 9 rivers. A, JinShaJiang River; B, Jialingjiang River; C, Daduhe River; D, Fujiang River; E, Qujiang River; F, Tuojiang River; G, Minjiang River; H, Yalongjiang River-Huanghe River. TCs, Tetracycline; OTC, oxytetracycline; CTC, chlortetracycline; TE, tetracycline; AMP, ampicillin; AMX, amoxicillin; CEX, cephalexin; CTX, cefotaxime; SAs, Sulfonamides; SD, sulfadiazine; SMZ, Sulfamethoxazole; FQs, Quinolones; ENR, enrofloxacin; LEV, levofloxacin; NOR, norfloxacin; MOX, moxifloxacin; LINs, Lincomide; CLI, clindamycin; CRPs, Amide alcohols; CAP, chloramphenicol; HSH, Hei Shui He Kou; DJYSWZ, Du Jiang Yan Shui Wen Zhan; MJDQ, Min Jiang Da Qiao; WGX, Wei Gu Xiang; RWGHK, Re Wu Gou He Kou; YX, Yin Xiu; WMQ, Wei Men Qiao; YLDK, Yue Lai Du Kou; MAS, Ma An Shan; YB, Yue Bo; WJ, Wen Jiang; LJG, Liang Jiang Gou; XJ, Xin Jin; HJP, Huang Jing Ping; LMT, Li Ma Tou; SGZ, San Gu Zhuang; SMZ, Shi Men Zi; HLK, Hu Lu Kou; NXDK, Na Xi Du Kou; SPY, Shou Pa Yan; LG, Luo Guo; HLQ, He Long Qiao; GGS, Gua Gong Shan; LJSDQ, Lin JiangShi Da Qiao; BJ, Ba Jiao; SHM, San Huang Miao; YSZ, Yin Shan Zhen; JXC, Jiao Xian Cun; HDDK, Huai De Du Kou; BMG, Ba Miao Gou; SX, Sha Xi; XDK, Xiao Du Kou; QPZ, Qing Ping Zhe; SSP, Shang Shi Pan; SLX, Sai Long Xiang; SBY, Shou Bang Yan; YJXC, Ya Jiang Xian Cheng; YLJKYa, Long Jiang Kou; MJQ, Mi Jia Qiao; BQ, Bai Qin; FTB, Fu Tian Ba; ZXC, Ze Xiu Cun.

This study analyzed nine typical rivers in Sichuan Province: the MinJiang River, DaDuHe River, JinSha River, TuoJiang River, JiaLingJiang River, QuJiang River, YalongJiang River, FuJiang River, and HuangHe River (S1 Fig). The monitoring sites across these rivers are represented by colorful dots on the map (S1 Fig). All 15 targeted antibiotics were successfully detected, with detection rates ranging from 2.38% for CTX to 83.33% for levofloxacin LEV (Table 4). The concentration of antibiotics varied across the rivers, with the highest levels observed in the MinJiang River (938 ng/L), followed by the TuoJiang River (177 ng/L) and the JiaLingJiang River (127 ng/L) (Fig 1). Notably, there was significant variability in antibiotic concentrations between different monitoring sites within the same river (Fig 1). Additionally, a clear trend of increasing antibiotic pollution was observed from the upper to the lower reaches of the rivers (S2 Fig). This trend was particularly pronounced in the JinSha River, MinJiang River, DaDuHe River, TuoJiang River, QuJiang River, YalongJiang River, and FuJiang River, where the cumulative antibiotic concentrations showed significant differences between the upper and middle reaches (S2 Fig, P<0.05). The distribution map of antibiotic concentrations (S2 Fig) indicates that the most heavily polluted monitoring sites were concentrated in the Sichuan Basin. These findings are consistent with the typical patterns of antibiotic use, industrial activities, and population density in the region’s rivers.

**Table 4:** Detection rate and concentration of 15 antibiotics in typical rivers (ng/L).

Categories	Antibiotic	Detection Rate(%)	Mean value	Median value	Highest Con	Lowest Con
TCs	OTC	45.23	60.04±167.66	51.93	938.4	5.25
	CTC	7.14	2.07±9.56	19.23	58.94	9.04
	TE	28.57	84.35±351.35	65.5	2233.71	7.63
β-lactams	AMP	38.09	1.23±3.52	2.1	22.61	1.25
	AMX	35.71	8.82±14.78	22.98	47.09	0.94
	CEX	14.28	5.56±17.93	23.81	88.65	5.9
	CTX	2.38	0.31±2	12.93	12.93	12.93
SAs	SD	38.09	1.71±3.83	2.03	15.56	0.41
	SMZ	21.42	1.44±5.1	2.97	31.47	0.85
QNs	ENR	80.95	24.42±31.58	26.67	170.56	0.29
	LEV	83.33	15.51±19.39	11.76	86.97	1.25
	NOR	42.85	24.87±34.69	52.43	127.42	7.88
	MOX	47.61	14.62±19.03	27.12	74	5.12
LINs	CLI	64.28	4.52±19.91	1.31	129.74	0.32
CRPs	CAP	30.95	25.48±90.63	32.6	577.46	3.43

Note: Con, concentration; TCs, Tetracycline; SAs,sulfonamides; QNs, quinolones; LINs, Lincoamide; CRPs, chloramphenicols; OTC, oxytetracycline; CTC, chlortetracycline; TE, tetracycline; AMP, ampicillin; AMX, amoxicillin; CEX, cephalexin; CTX, cefotaxime; SAs, Sulfonamides; SD, sulfadiazine; SMZ, Sulfamethoxazole; FQs, Quinolones; ENR, enrofloxacin; LEV, levofloxacin; NOR, norfloxacin; MOX, moxifloxacin; LINs, Lincomide; CLI, clindamycin; CRPs, Amide alcohols; CAP, chloramphenicol.

### Antimicrobial resistance of *E*. *coli* in rivers

**Fig 2:**
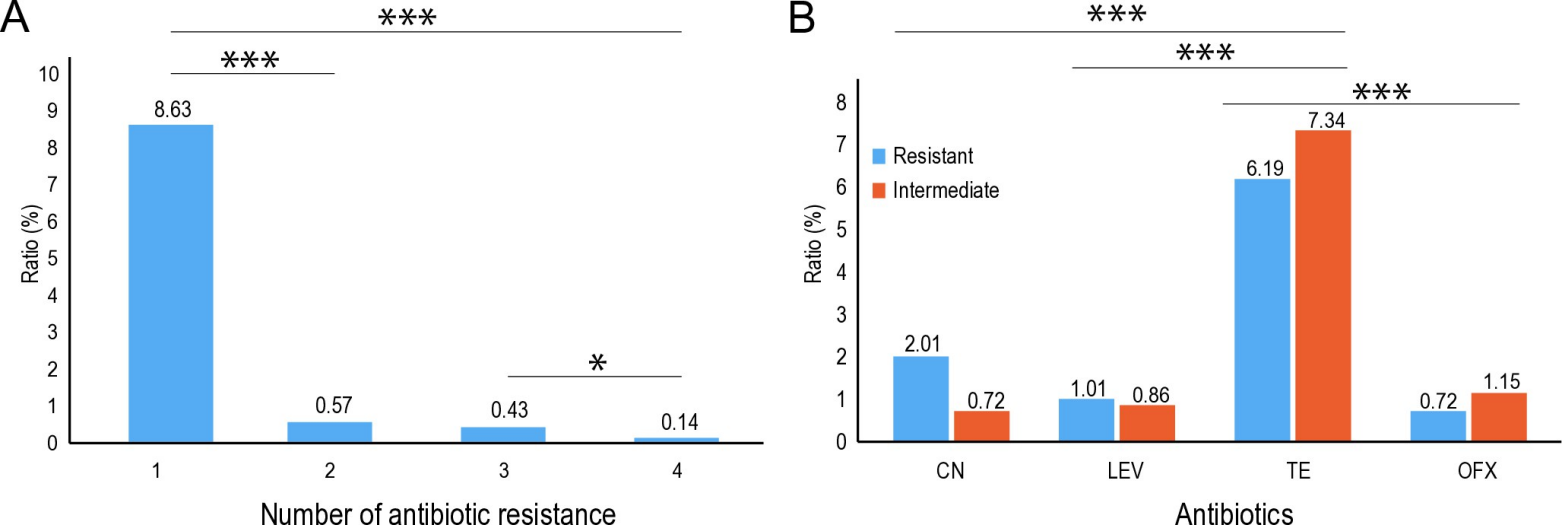
Resistance of *E*. *coli* to four antibiotics. A, Resistance of 695 *E*. *coli* to four antibiotics. B, The proportion of different antibiotic-resistant bacteria in the population of *E*. *coli*. CN, Gentamicin; LEV, Levofloxacin; TE, Tetracycline; OFX: Ofloxacin. *P<0.05, ***P<0.001.

Antibiotic pollution poses significant risks not only to ecological balance but also to human health. Prolonged antibiotic residue in water bodies can lead to the development of drug-resistant bacteria, complicating medical treatments and potentially endangering lives. To assess the level of antibiotic resistance, 695 *E*. *coli* strains were isolated from typical rivers and exposed to four different antibiotics: CN, LEV, TE, and OFX. The results showed that 9.77% of *E*. *coli* strains were resistant to at least one antibiotic, with the proportion of resistant strains decreasing as antibiotic treatment intensity increased (Fig 2A). Specifically, 8.63% were resistant to one antibiotic, 0.57% to two, and multidrug-resistant (MDR) bacteria constituted only 0.43% of the isolates (Fig 2A). Notably, only one strain (SHM-14) exhibited resistance to all four antibiotics (CN, LEV, TE, and OFX). Additionally, *E*. *coli* displayed varying levels of resistance to these antibiotics (Table 5). E. coli exhibited the highest resistance to tetracycline, with a 6.1% resistance ratio and a 7.34% intermediate resistance rate (Fig 2B). This high resistance to TE may be attributed to its prevalence in the rivers studied, explaining its status as the antibiotic with the highest *E*. *coli* resistance. Furthermore, it was observed that antibiotic resistance levels were highest in the YaLongJiang River and lowest in the HuangHe river (S3 Fig).

**Table 5:** Drug resistance rate of *E*. *coli* in 9 typical river systems (%).

Rivers	CN	LEV	TE	OFX
Minjiang River(n = 176)	1.14	1.7	5.11	0. 57
JinShaJiang River(n = 120)	2.52	0.83	6.67	0.83
DaDuHe River(n = 51)	0	0	11.17	0
TuoJiang River(n = 98)	4.08	2.04	7.14	1.02
JiaLingJiang River(n = 92)	1.08	0	7.78	0
QuJiang River(n = 31)	0	0	6.45	0
FuJiang River(n = 62)	0	0	8.06	0
YaLongJiang River(n = 37)	10.08	2.7	5.4	5.4
HuangHe River(n = 28)	0	0	0	0

Note: CN, Gentamicin; LEV, Levofloxacin; TE, Tetracycline; OFX: Ofloxacin.

### Risk assessment of typical rivers

**Fig 3:**
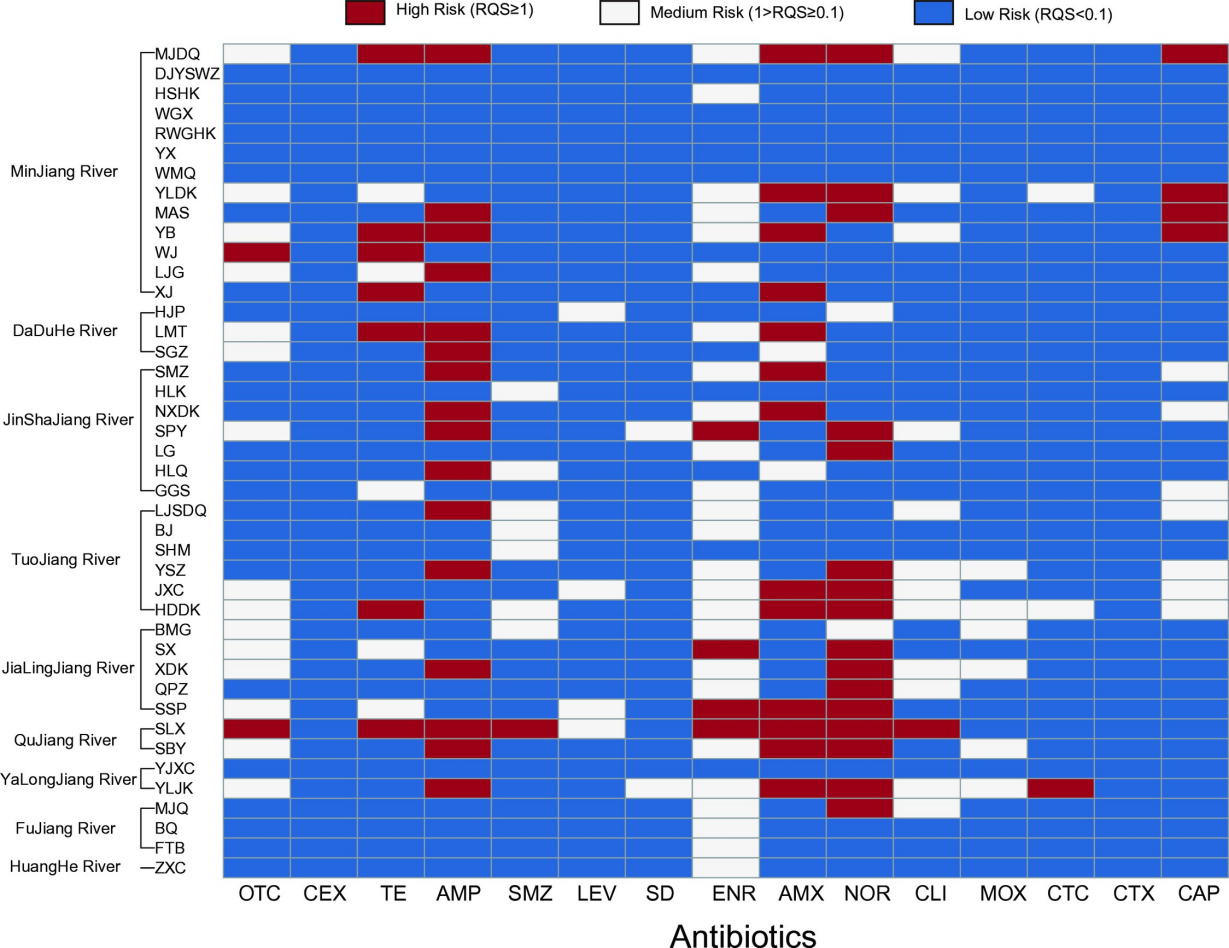
Risk assessment of antibiotic at monitoring. TCs, Tetracycline; OTC, oxytetracycline; CTC, chlortetracycline; TE, tetracycline; AMP, ampicillin; AMX, amoxicillin; CEX, cephalexin; CTX, cefotaxime; SAs, Sulfonamides; SD, sulfadiazine; SMZ, Sulfamethoxazole; FQs, Quinolones; ENR, enrofloxacin; LEV, levofloxacin; NOR, norfloxacin; MOX, moxifloxacin; LINs, Lincomide; CLI, clindamycin; CRPs, Amide alcohols; CAP, chloramphenicol.

The distribution of antibiotics and drug-resistant bacteria in rivers can significantly disrupt the ecological balance, necessitating a thorough ecological risk assessment of antibiotic residues in typical rivers across Sichuan. Bacteria, algae, and water fleas were selected as key indicators for this analysis due to their prevalence in these river ecosystems [30]. As shown in Fig 3, antibiotic residues at 26 of the 42 monitoring sites posed a high ecological risk, while 10 sites were categorized as medium risk. The most significant environmental risk factors within the Sichuan River system were NOR, amoxicillin (AMX), ampicillin (AMP), and tetracycline (TE). NOR and AMP were the most prevalent, each contributing 38.09% to the total risk, followed by AMX (30.95%), TE (16.67%), ENR and CAP (each 9.52%), and OTC (4.76%). At the lower end, CTC, CLI, and SMZ posed the least risk (2.38%). The RQEcotox values for the 15 antibiotics analyzed ranged from 0 to 72.94. CTX posed the lowest ecological risk, with the smallest mean RQEcotox value (0.001) among the 42 monitoring sites. In contrast, AMP and AMX displayed the highest ecological risks, with average RQEcotox values of 4.066 and 2.433, respectively. Additionally, the ecological risk varied across the different rivers studied. The HuangHe and FuJiang Rivers exhibited the lowest ecological risks (Fig 3), while the other seven rivers showed significant contamination. Moreover, there was a marked difference in risk levels between upstream and downstream regions. The middle and downstream areas, where most monitoring sites were classified as high risk, exhibited the greatest ecological threats. This pattern aligns with the higher population density in the basin’s middle and lower reaches, indicating a strong correlation between ecological risk and human activity.

### Microbial diversity and community structure of Minjiang River

**Fig 4:**
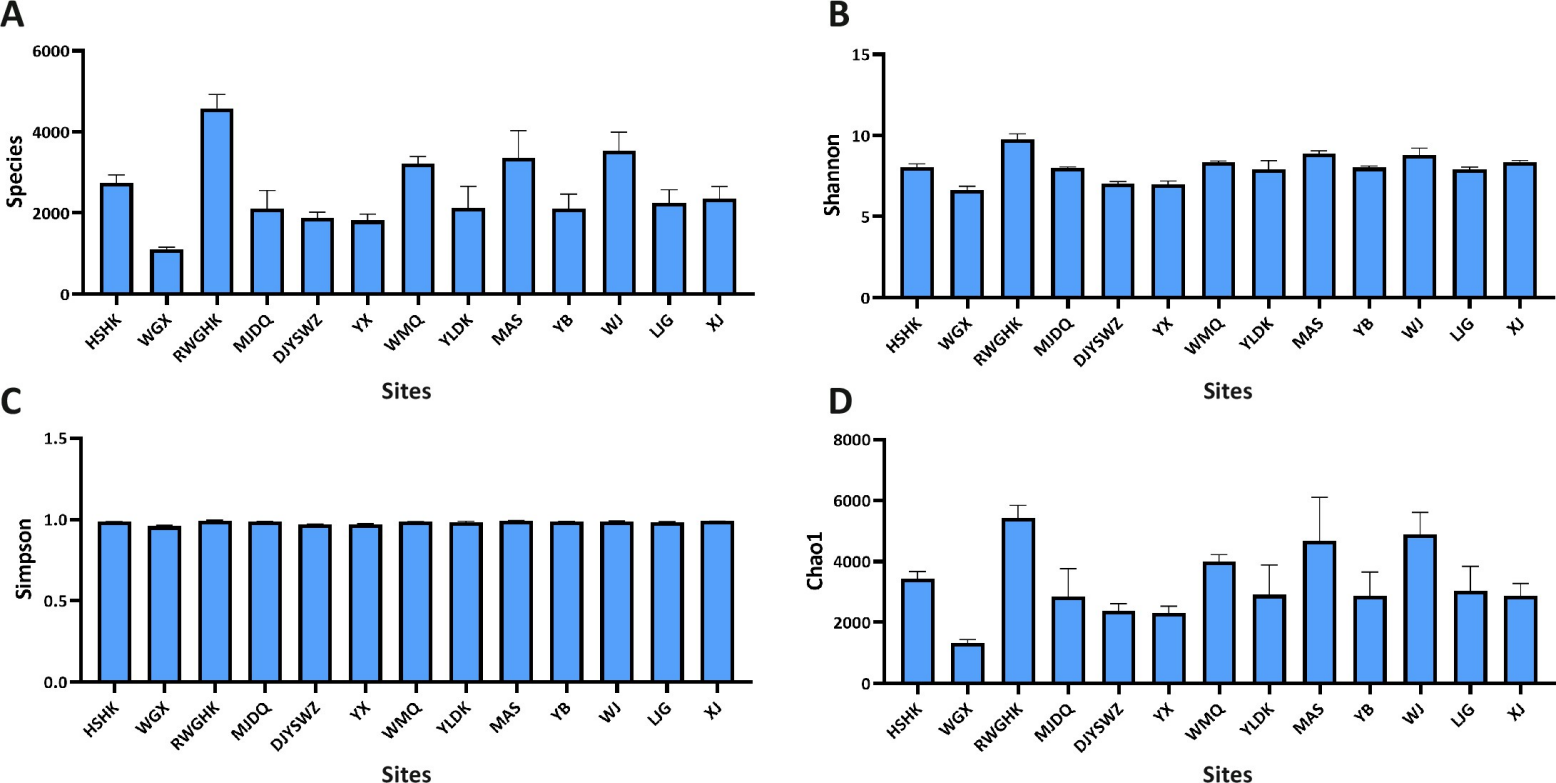
ɑ-diversity data of monitoring sites in Minjiang River. A, Species value. B, Shannon value. C, Simpson value. D, Chao1 value.

**Fig 5:**
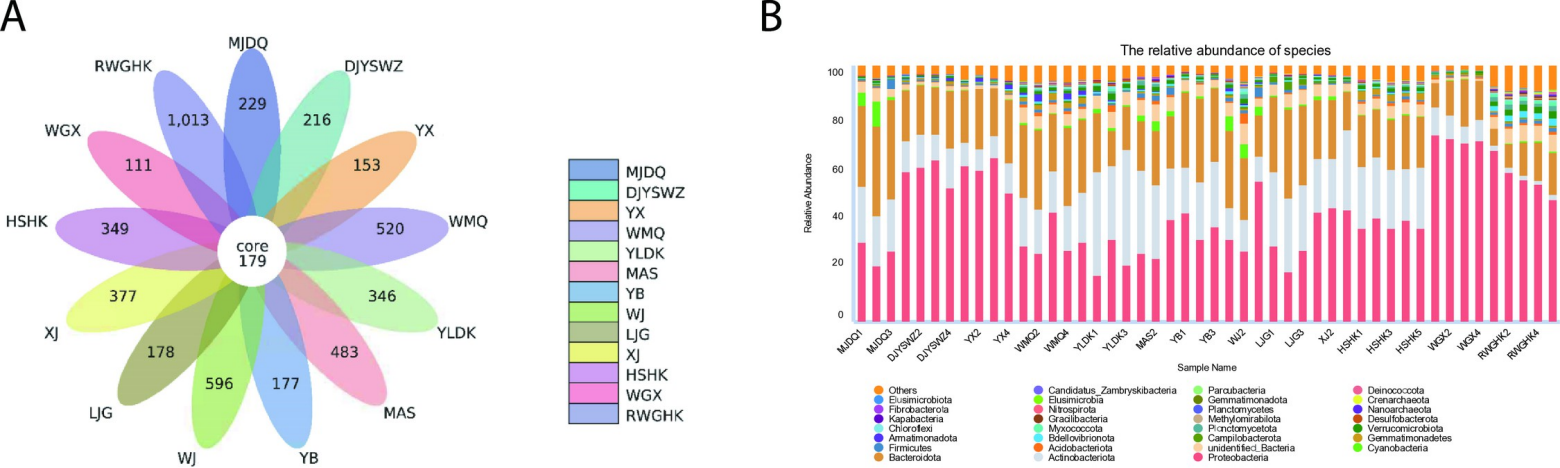
Operational taxonomic units and taxonomic composition distribution of MinJiang River. A, Venn diagrams that showing the shared and distinct OTUs in sites of Minjiang River. B, Taxonomic composition distribution histograms in monitoring sites at phylum. OTUs, operational taxonomic units.

To further investigate the impact of antibiotics on microbial diversity, microorganisms from 51 water samples across 13 monitoring sites were sequenced. The Re Wu Gou He Kou (RWGHK) located in upstream of Minjiang river,RWGHK monitoring site exhibited the highest indices of microbial diversity and richness, with Species (4585), Shannon (9.75), Simpson (9.94), and Chao 1 (5427) indices (Fig 4A–4D), indicating that RWGHK had the most favorable ecological environment. In contrast, the WGX site recorded the lowest values, with Species (1098), Shannon (6.66), Simpson (0.95), and Chao 1 (1332) indices, suggesting a less diverse and rich microbial community. Additionally, the similarity of microbial communities across the sites was assessed. A total of 179 operational taxonomic units (OTUs) were shared among the 13 monitoring sites (Fig 5A), with each site also harboring unique OTUs. Proteobacteria emerged as the dominant phylum across all samples, with an average proportion ranging from 24% to 71.25%, followed by Actinobacteria (1.8% to 38.17%) and Bacteroidetes (12.04% to 35.19%) (Fig 5B). Our study demonstrated that antibiotic pollution can reduce the abundance of Proteobacteria, as sites with lower antibiotic concentrations (Du Jiang Yan Shui Wen Zhan in MinJiang (DJYSWZ), Yin Xiu (YX) in MinJiang, Wei Gu Xiang (WGX) in MinJiang, RWGHK exhibited a Proteobacteria proportion exceeding 50%.

**Fig 6:**
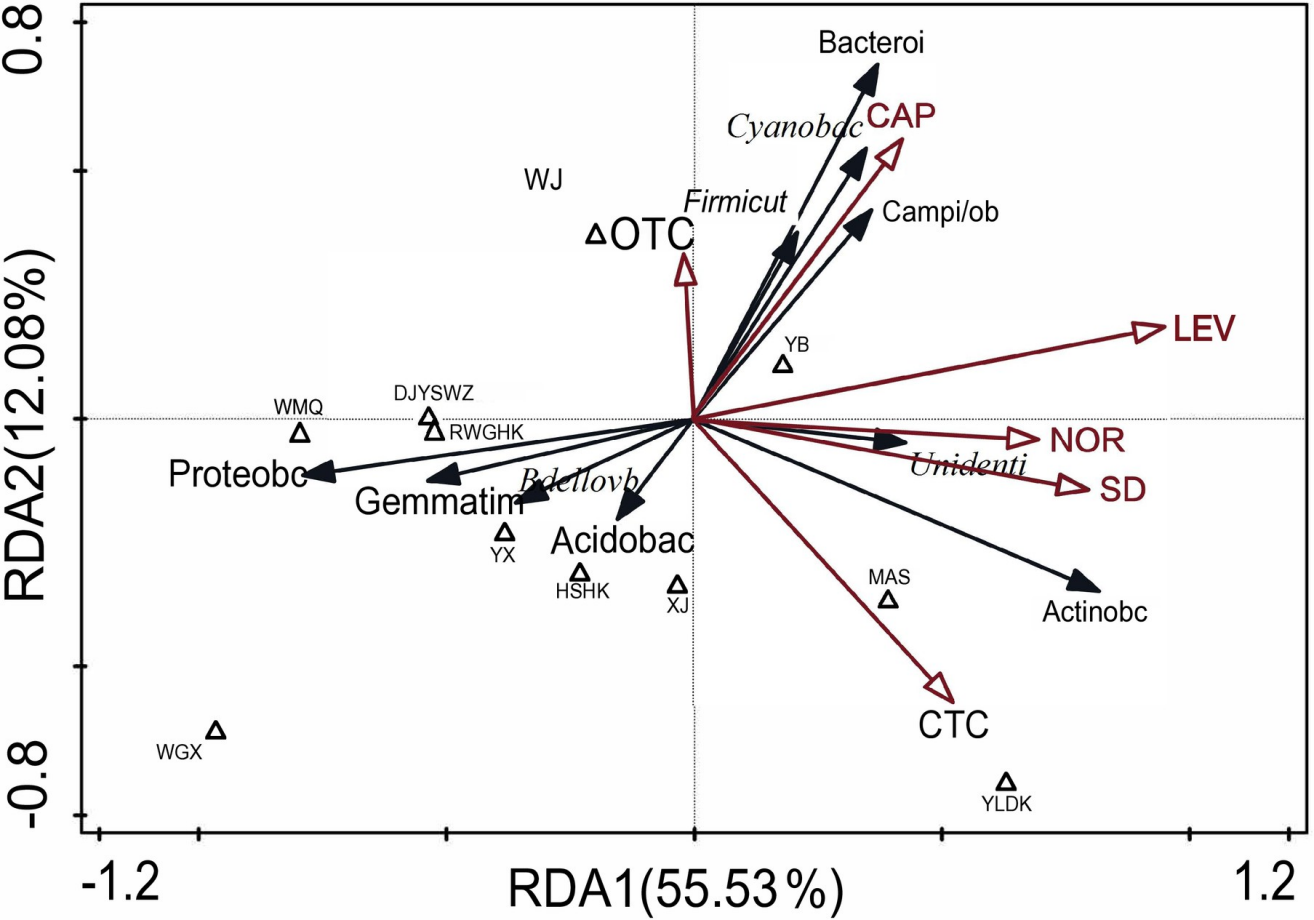
Redundancy analysis (RDA) of antibiotics residual and bacterial communities. Triangle represents each sample. Antibiotics and bacterial (phylum) were indicated by hollow arrows and Solid arrows.

To explore the relationship between antibiotics and microbial communities in greater detail, RDA was conducted (Fig 6). Six antibiotics (OTC, CAP, LEV, NOR, SD, and CTC) were selected for the analysis based on the results from the Monte Carlo test. The RDA results revealed that the microbial communities at Wei Men Qiao located in MinJiang River (WMQ), DJYSWZ, and RWGHK were similar, likely due to the close proximity of these monitoring sites, which led to similar carbon and nitrogen content in the environment. Additionally, LEV, SD, NOR, CTC, and CAP were identified as significant influencers of the microbial community structure. The negative association was found between *Proteobacteria* and LEV[-0.756_Proteobacteria_(p<0.01)] and SD[-0.583_Proteobacteria_(p<0.05)]. On the contrary, LEV[0.744_Actinobacteriota_ (p<0.01)], SD[0.71_Actinobacteriota_ (p<0.01)], NOR[0.563_Actinobacteriota_ (p<0.05)] and CTC[0.594_Actinobacteriota_ (p<0.05)] present positive association to *Actinobacteriota*. Besides, CAP was positively associated to the *Bacteroidota* [0.555_Bacteroidota_ (p<0.01)] and *Cyanobacteria*[0.694_Cyanobacteria_ (p<0.01)]. Most antibiotics present positive association to the microbial community.

## Discussion

Antibiotic pollution in rivers is a significant issue that poses risks not only to human health but also to the environment. This study investigated the levels of antibiotic residues and the associated ecological risks in typical rivers of Sichuan. Additionally, we assessed the effects of these residues on *E*. *coli* antibiotic resistance and the broader microbial community within these water systems. Our findings revealed the presence of all 15 antibiotics across nine rivers in Sichuan, with concentrations ranging from 0.29 ng/L to 2233.71 ng/L. FQs were identified as the predominant pollutants. Furthermore, 9.77% of *E*. *coli* strains exhibited drug resistance, with more than 5.8% showing MDR. Ecological risk assessment indicated that 26 out of 42 monitoring sites were classified as high risk, with NOR, AMP, AMX, and TE identified as the most significant risk factors. A strong correlation between microbial community structure and antibiotics such as LEV, SD, NOR, TCT, and CAP was also observed, suggesting these antibiotics may impact the ecological balance, particularly in the MinJiang River.

Antibiotics, particularly TCs and FQs, have been widely used in both human and veterinary medicine [31–33]. Our study detected significant levels of these antibiotics in the rivers of Sichuan Province, with concentrations ranging from 0.29 ng/L to 2233.71 ng/L. When compared to global data, these levels are relatively high. For instance, studies on the Litani River in the Lebanon reported antibiotic concentrations ranging from 107 to 250 ng/L [34], while the median concentrations of SMX, TMP, AMX, and AETM in the water of Africa were 286, 122, 78.0, and 86.0 ng/L, respectively [35]. The detected concentrations in the MinJiang River, which reached 2233.71 ng/L, suggest a higher burden of antibiotic pollution in Sichuan compared to many international water bodies.

This broad range highlights the pervasive nature of antibiotic pollution in these water bodies. Our findings are consistent with previous studies that have documented the presence of antibiotics in various environmental settings, including rivers. For example, Zhang et al. (2015) reported similar levels of antibiotic contamination in major rivers across China, where the presence of TCs and FQs was linked to intensive agricultural and aquaculture activities [36]. Similarly, Lei et al. observed high concentrations of these antibiotics in the Haihe River, which they attributed to both agricultural runoff and wastewater discharge from pharmaceutical factories [37]. In our study, the detection rates for TCs and FQs were significant, though slightly lower than those reported in other regions such as the Yangtze River, where the pollution levels were higher due to the more extensive industrial and agricultural activities in that area [38]. The lower detection rates in our study may reflect the specific environmental conditions in Sichuan, including the adsorption properties of local sediments, as well as recent policy efforts to reduce antibiotic use in agriculture [39].

Given the widespread use of antibiotics in both human healthcare and animal agriculture, it is vital to explore the connection between antibiotic concentrations in the environment and the size of the populations that are contributing to this usage. The correlation between population density and environmental antibiotic levels is not just a reflection of consumption patterns but also an indicator of the potential for increased antibiotic resistance in these areas. Larger populations, especially in regions with intensive agricultural practices, tend to exert greater pressure on local ecosystems through the release of antibiotic residues. This, in turn, exacerbates the risks of resistance development, underscoring the need for targeted interventions in areas with high population densities and antibiotic usage. Our study found that antibiotic concentrations varied significantly between different rivers, likely influenced by population density. For example, the MinJiang River exhibited much higher antibiotic concentrations than the HuangHe River, which could be explained by the permanent population of 25 million along the MinJiang River [40] compared to only 0.4 million along the HuangHe River [41]. Higher population densities typically result in greater antibiotic usage, leading to higher concentrations of antibiotic residues in rivers. Additionally, within the same river, antibiotic concentrations varied between monitoring sites, a phenomenon also observed in China’s YongJiang River [42] and FenHe River [43]. These studies indicated that the higher population density downstream could contribute to increased antibiotic concentrations. Another factor influencing antibiotic usage is animal husbandry, which is well-developed in the Sichuan Basin due to its unique geographical environment. Approximately 85% of Sichuan’s population resides in this basin, leading to extensive farming activities. However, less than 50% of these activities are intensive, meaning that over 50% are carried out by small-scale or individual farmers, who may overuse antibiotic feed due to regulatory challenges. Furthermore, the improper disposal of agricultural waste, often directly discharged into the environment, contributes to the presence of residual antibiotics in water. These findings suggest that reducing antibiotic concentrations in wastewater from livestock and aquaculture could be the most direct and effective strategy for mitigating antibiotic contamination. Globally, similar trends have been observed in regions with dense populations and intensive agriculture. For example, the Msunduzi River in Eastern South Africa reported antibiotic concentrations as high as 1290 ng/L [44], while the Seine River in France exhibited levels up to 1210 ng/L [45]. These figures align with our findings, indicating that antibiotic pollution in Sichuan shares similarities with other heavily populated and agriculturally intensive regions worldwide.

In our study, we identified that antibiotic pollution poses significant risks to both ecological balance and human health. Prolonged exposure to antibiotic residues in water bodies can foster the development of drug-resistant bacteria, which complicates medical treatments and poses a potential threat to human life. The mechanisms through which *E*. *coli* acquires resistance are primarily through horizontal gene transfer (HGT), spontaneous mutations, and plasmid-mediated resistance. HGT allows *E*. *coli* to acquire resistance genes from other bacteria in the environment, especially in polluted rivers where selective pressure is high. Spontaneous mutations within the *E*. *coli* genome can lead to antibiotic resistance by altering target sites, increasing efflux activity, or decreasing membrane permeability to antibiotics. Additionally, plasmid-mediated resistance facilitates the transfer of resistance genes across different bacterial strains and species, contributing significantly to the spread of MDR. Our results showed that 9.77% of *E*. *coli* strains exhibited resistance to at least one antibiotic, with over 5.8% demonstrating MDR. These findings underscore the extensive nature of antibiotic pollution and its profound impact on microbial communities. The mechanisms through which *E*. *coli* acquires resistance are primarily through horizontal gene transfer (HGT), spontaneous mutations, and plasmid-mediated resistance. HGT allows *E*. *coli* to acquire resistance genes from other bacteria in the environment, especially in polluted rivers where selective pressure is high. Spontaneous mutations within the *E*. *coli* genome can lead to antibiotic resistance by altering target sites, increasing efflux activity, or decreasing membrane permeability to antibiotics. Additionally, plasmid-mediated resistance facilitates the transfer of resistance genes across different bacterial strains and species, contributing significantly to the spread of MDR. When compared with other studies, our findings are consistent with existing literature on antibiotic pollution and the emergence of resistance. For instance, Su et al. reported a high prevalence of MDR *E*. *coli* in the Dongjiang River, with an MDR rate of 87.5% [46]. Similarly, research by Zheng et al. on the Jiulong River revealed that severe antibiotic pollution significantly contributed to the development of bacterial resistance, with an MDR rate of 70.6% [47]. However, the MDR rate in our study was slightly lower, which could be attributed to the specific types of antibiotics and the range of detection methods we used, or it may reflect the recent strengthening of environmental protection policies in Sichuan Province, such as the prohibition of antibiotics as feed additives and stricter sewage discharge standards. Our findings also demonstrated that antibiotic pollution in rivers can disrupt ecological balance, with AMP and AMX presenting the highest ecological risks. Previous research has shown that AMP and AMX can be toxic to *Microcystis aeruginosa* at very low concentrations (3.7 ng/L and 0.31 ng/L) [48, 49]. Despite the short half-life of β-lactam antibiotics in aquatic environments (1–2 hours), these data highlight the need for careful consideration of the ecological risks posed by these substances [50]. Moreover, our study found that the ecological risks associated with antibiotic pollution were linked to changes in microbial community structure, with most antibiotics showing positive associations with microbial communities. Similar findings have been reported in other studies, where low concentrations of LEV were found to inhibit Azospirillum and impair the nitrogen-fixing ability of microorganisms [51]. Likewise, Vanesa et al. reported that CTC was toxic to soil microbial communities in France [52]. Overall, these results suggest that residual antibiotics can act as environmental selective pressures, altering microbial community structure and composition.

### Limitations

While this study provides valuable insights into the distribution of antibiotic residues, ecological risks, and phenotypic antibiotic resistance of *E*. *coli* in typical rivers of Sichuan Province, it does have certain limitations. Firstly, the study primarily focused on phenotypic resistance patterns and ecological risk assessment. Although molecular analysis of *E*. *coli* would provide a more comprehensive understanding of the resistome and the underlying genetic mechanisms driving antibiotic resistance, it was beyond the scope of this study due to resource and time constraints. Secondly, while our sampling covered both wet and dry seasons, temporal variations in antibiotic resistance genes (ARGs) and their correlation with environmental factors could be further explored through long-term monitoring and seasonal molecular analysis. Future research should incorporate advanced molecular techniques, such as whole-genome sequencing and metagenomics, to investigate the resistome in detail and better understand the horizontal gene transfer mechanisms contributing to antibiotic resistance in aquatic ecosystems. We believe these limitations do not diminish the value of our findings but instead highlight opportunities for further research in this critical area.

## Conclusion

Our study highlights the critical issue of antibiotic pollution in rivers and its far-reaching implications for both environmental health and public safety. By systematically analyzing antibiotic residues and their impact on microbial communities and antibiotic resistance, our research underscores the urgent need for more stringent regulations and effective management strategies to control antibiotic contamination. The findings emphasize the importance of addressing pollution sources, particularly in regions with high human activity and agricultural practices, to protect aquatic ecosystems and prevent the spread of antibiotic-resistant bacteria. This work contributes valuable insights into the complex interactions between antibiotics and microbial ecosystems, offering a foundation for future research and policy development aimed at safeguarding environmental and human health.

## Supporting information

S1 Fig The map of 9 typical rivers and monitoring sites in Sichuan. (DOCX)

S2 Fig Distribution of antibiotics residue (total concentration) in typical rivers water of Sichuan. (DOCX)

S3 Fig The spatial distribution maps of resistance patterns. (DOCX)

S1 Table Original data. (XLSX)

S1 File (XLSX)
