# Supplementary material for: Risk assessment of antibiotic residues and resistance profile of E. coli in typical rivers of Sichuan, China
Source: PLoS One. 2025 Feb 11;20(2):e0306161. doi: 10.1371/journal.pone.0306161 (PMC11813091; doi:10.1371/journal.pone.0306161)

Supplementary

**Figure legends:**

Figure S1. The map of 9 typical rivers and monitoring sites in Sichuan

Fig S1.


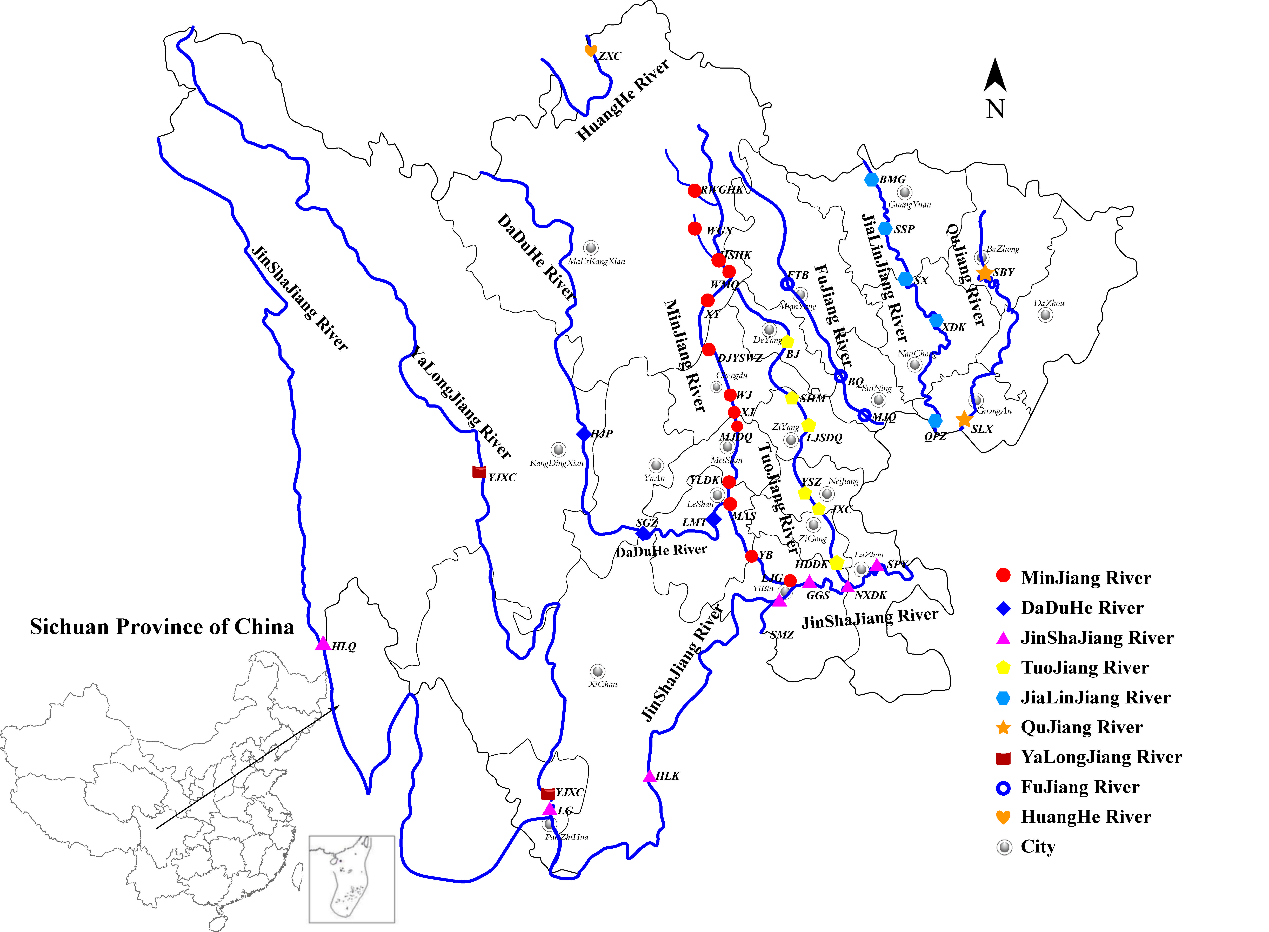

Supplement: S1 Fig — (DOCX) [file pone.0306161.s001.docx]
